# Use of Intraoperative Methylene Blue Spray for Identification and Preservation of Vital Structures in Thyroidectomy: A Prospective Comparative Study

**DOI:** 10.7759/cureus.104801

**Published:** 2026-03-06

**Authors:** Muhammad Ammar Bashir, Nakash Ahsan, Rashid Mehmood, Muhammad Azam Tayyab, Muhammad Usman Javed, Shabbar H Changazi, Samiullah Bhatti

**Affiliations:** 1 General Surgery, Islam Teaching Hospital, Sialkot, Sialkot, PAK; 2 General Surgery, Gujranwala Medical College Teaching Hospital, Gujranwala, PAK; 3 General Surgery, Tehsil Headquarters (THQ) Hospital, Daska, Daska, PAK; 4 General Surgery, District Headquarters (DHQ) Teaching Hospital, Gujranwala, Gujranwala, PAK; 5 General Surgery, Tehsil Headquarters (THQ) Hospital, Renala Khurd, Renala Khurd, PAK; 6 General Surgery, Provincial Headquarter Teaching Hospital, Gilgit, Gilgit, PAK; 7 Surgery, Services Hospital Lahore, Lahore, PAK

**Keywords:** goiter, hypocalcemia, intraoperative identification, methylene blue, nerve preservation, parathyroid glands, randomized controlled trial, recurrent laryngeal nerve injury, surgical technique, thyroidectomy

## Abstract

Introduction

Although thyroidectomy is relatively safe, iatrogenic injury to the recurrent laryngeal nerve (RLN) and parathyroid glands (PGs) can cause major morbidity. Conventional visual identification can be challenging. This study aims to determine the utility of intraoperative methylene blue (MB) spray for the localization and preservation of these structures.

Methods

A comparative interventional study was conducted with 136 patients undergoing thyroidectomy for benign multinodular goitre. Patients were divided into an experimental group (topical MB spray, n = 68) and a control group (conventional dissection, n = 68). Primary outcomes were the frequency of RLN injury, assessed via postoperative laryngoscopy, and mean postoperative serum calcium levels measured 72 hours after the surgery.

Results

The mean serum calcium level on the first postoperative day was significantly higher in the group of MB spray (8.87 ±0.45 mg/dL) than that of the control group (8.21 ± 0.33 mg/dL; p < 0.001). There were no cases of RLN injury in the MB spray group compared with 11.8% in the control group (p = 0.006). These significant differences persisted across all patient subgroups.

Conclusion

Intraoperative MB spraying is helpful for accurate identification and preservation of RLNs and PGs during thyroidectomy. This technique results in higher postoperative calcium levels and a markedly reduced incidence of RLN injury, advocating for its routine use in thyroid surgery.

## Introduction

Thyroidectomy is a frequently performed procedure for thyroid diseases with low mortality. However, postoperative complications, especially those that affect patients' quality of life, remain challenging. The most common iatrogenic injuries are those involving the recurrent laryngeal nerve (RLN) and the parathyroid glands (PGs), as both structures are closely anatomically related to the thyroid gland [1,2].

When the RLN is damaged, it can lead to paralysis of the vocal cord and different levels of hoarseness, aspiration, and even lethal airway obstruction (if bilaterally injured). Parathyroid damage or accidental removal causes hypocalcaemia, which may be transient or permanent, requiring lifelong medical therapy. These issues emphasize the crucial nature of appropriate intraoperative localisation [3,4].

Conventional surgery relies on meticulous visual dissection based on anatomical landmarks. However, this technique is not entirely fail-safe, particularly in the presence of anatomical variation, re-operation, or a large pathological gland, and it can be difficult for novice surgeons. Therefore, adjuncts to aid visualization are helpful [5,6].

Methylene blue (MB), a versatile synthetic dye widely used in clinical medicine, has been proposed as a tool to facilitate this identification. When used as an intraoperative spray, MB differentially stains tissues, allowing vital structures to be more easily distinguished from the surrounding thyroid parenchyma. While international studies have suggested that MB spraying may improve the identification and preservation of these structures, there is a lack of localized clinical evidence within our specific patient population [7,8]. This comparative interventional study aimed to compare the outcomes of thyroidectomy with and without intraoperative MB spraying.

## Materials and methods

This was a prospective comparative interventional study conducted at the Department of General Surgery, Services Institute of Medical Science, Lahore, Pakistan, from December 2020 to November 2025. Ethical approval was obtained, and written informed consent was secured from all participants. The trial is registered on ClinicalTrials.gov under the identifier NCT07395388. The required sample size was calculated as 136 patients (68 per group). This calculation assumed an RLN injury rate of 0% in the MB group compared to 8.6% in the control group, with 80% power and a 5% significance level.

Patients aged 18-60 years with benign multinodular goitre scheduled for thyroidectomy were included. Exclusion criteria included recurrent or metastatic disease, preoperative vocal cord dysfunction, coagulopathy, significant hepatic or renal impairment, known MB allergy, and re-operative surgery. Participants were randomly allocated to either the MB spray group or the conventional surgery control group using a computer-generated random number sequence.

All procedures were completed under general anaesthesia by a single surgical team via standard collar neck incision. The experimental group was sprayed with 0.5 mL of MB onto the mobilized thyroid lobe after retraction, and the surrounding tissues. Blue staining was washed out of the PGs, which returned to a yellowish colour within three minutes (range 2-5 minutes), while thyroid tissue remained stained for up to 15 minutes (range 12-22 minutes). This differential clearance helped to identify before meticulous dissection. Visual identification and dissection were performed using the control group.

RLN injury was assessed by the attending anesthetist immediately after extubation using indirect laryngoscopy. The assessor was blinded to the patient's group allocation to minimize detection bias. RLN injury was defined as the absence of vocal cord movement. Patients with identified RLN injury underwent follow-up indirect laryngoscopy at three, six, and 12 months postoperatively to assess recovery of vocal cord function. The serum calcium level was measured 72 hours after surgery. Patients with low serum calcium levels at 72 hours were monitored biochemically and followed up for three months. Demographic information, type of surgery, and outcomes were documented. Statistical processing was carried out by means of IBM SPSS Statistics for Windows, Version 21 (Released 2012; IBM Corp., Armonk, New York, United States). Continuous variables were analyzed with an independent samples t-test, and categorical variables by chi-square or Fisher’s exact test, with p ≤ 0.05 defined as statistically significant. Subgroup analyses were performed based on age, gender, BMI, and type of surgery.

## Results

A total of 136 patients were included. The mean age of the study population was 36.6 ± 13.3 years. The cohort was predominantly female (80.1%), yielding a male-to-female ratio of 1:4. Total thyroidectomy was the most frequent procedure (66.2%, n = 90). Both the MB spray and the conventional surgery groups had similar patient populations in terms of age, gender distribution, BMI, and type of surgery. This balance ensures that any differences in outcomes are likely due to the treatment rather than other factors. The demographic information is depicted in Table 1.

**Table 1:** Baseline Demographic and Clinical Characteristics Data are presented as mean ± standard deviation (SD) or n (%). P-values were calculated using the independent samples t-test (t) for continuous variables and the chi-square test (χ^2^) for categorical variables.

Characteristic	Methylene Blue Group (n = 68)	Control Group (n = 68)	Test Statistic	p-value
Age (years), Mean ± SD	36.5 ± 13.1	36.7 ± 13.6	t = 0.083	0.934
Age Category, n (%)				
<25 years	19 (27.9)	20 (29.4)	χ^2^ = 0.126	0.939
25-50 years	39 (57.4)	37 (54.4)		
>50 years	10 (14.7)	11 (16.2)		
Gender, n (%)				
Male	14 (20.6)	13 (19.1)	χ^2^ = 0.046	0.830
Female	54 (79.4)	55 (80.9)		
BMI (kg/m^2^), Mean ± SD	28.6 ± 3.2	28.4 ± 3.3	t = 0.359	0.720
BMI Category, n (%)				
Non-obese	42 (61.8)	43 (63.2)	χ^2^ = 0.032	0.859
Obese	26 (38.2)	25 (36.8)		
Type of Surgery, n (%)				
Total Thyroidectomy	45 (66.2)	45 (66.2)	χ^2^ = 0.107	0.948
Lobectomy + Isthmusectomy	16 (23.5)	17 (25.0)		
Near Total Thyroidectomy	7 (10.3)	6 (8.8)		

The mean postoperative serum calcium value was significantly higher in the MB spray group (8.87 ± 0.45 mg/dL) than in the control group (8.21 ± 0.33 mg/dL; p < 0.001). This difference remained statistically significant across all stratified subgroups, including age, gender, and surgery type. Stratified analysis confirmed that this benefit was consistent and statistically significant across all patient subgroups, including age categories (<25 years: p < 0.001; 25-50 years: p < 0.001; >50 years: p = 0.002), gender (male: p < 0.001; female: p < 0.001), BMI categories (non-obese: p < 0.001; obese: p < 0.001), and type of surgery (total thyroidectomy: p < 0.001; lobectomy: p < 0.001; near-total: p = 0.034).

RLN paralysis occurred in eight (5.9%) of patients overall. Significantly, all injuries were in the control (11.8%), as compared with no injuries (0%) in the MB spray group (p = 0.006). This strong protective effect of MB spray was observed in most subgroups (Table 2).

**Table 2:** Stratified Analysis of Recurrent Laryngeal Nerve Injury Incidence All p-values were calculated using Fisher's exact test. MB: methylene blue; CI: confidence interval

Subgroup	MB Spray Group (n = 68)	Control Group (n = 68)	Difference (95% CI)	p-value
Overall	0 (0.0%)	8 (11.8%)	11.8% (4.2% to 19.4%)	0.006
Age				
<25 years	0/19 (0.0%)	2/20 (10.0%)	10.0% (-3.1% to 23.1%)	0.487
25-50 years	0/39 (0.0%)	5/37 (13.5%)	13.5% (2.5% to 24.5%)	0.024
>50 years	0/10 (0.0%)	1/11 (9.1%)	9.1% (-7.9% to 26.1%)	1.000
Gender				
Male	0/14 (0.0%)	1/13 (7.7%)	7.7% (-6.8% to 22.2%)	0.481
Female	0/54 (0.0%)	7/55 (12.7%)	12.7% (3.9% to 21.5%)	0.013
BMI Category				
Non-obese	0/42 (0.0%)	5/43 (11.6%)	11.6% (2.0% to 21.2%)	0.055
Obese	0/26 (0.0%)	3/25 (12.0%)	12.0% (-0.8% to 24.8%)	0.110
Type of Surgery				
Total Thyroidectomy	0/45 (0.0%)	5/45 (11.1%)	11.1% (1.9% to 20.3%)	0.056
Lobectomy + Isthmusectomy	0/16 (0.0%)	2/17 (11.8%)	11.8% (-3.7% to 27.3%)	0.485
Near Total Thyroidectomy	0/7 (0.0%)	1/6 (16.7%)	16.7% (-13.2% to 46.6%)	0.462

## Discussion

This comparative study shows that prophylactic MB spray topically applied during thyroidectomy greatly enhances patients' recovery. The method achieved a 0% rate of RLN injury and showed significantly higher postoperative serum calcium levels than conventional visual dissection alone. Our results firmly support the use of MB spray as a simple, potent supplement for safe surgery.

Our findings align with the emerging international literature. Farghaly et al. also showed a dramatic decrease in RLN injury and a higher postoperative calcium level with MB spray [9]. Sebaey et al. also noted a lower rate of transient vocal cord palsy in their MB group (0% vs. 8.6%) [10]. This occurs because of the differential pharmacokinetics of MB: parathyroid tissue rapidly takes up and washes out the dye, becoming yellow within a few minutes, while thyroid tissue, fat, and muscle retain the blue colour for longer periods, creating a marked visual contrast that facilitates identification [11].

Of note, in the control group, all eight cases of RLN injury manifested as unilateral vocal cord paralysis. After this review, four cases were resolved during the first three months, and another two cases were resolved over the next three months of follow-up. Nevertheless, two patients demonstrated ongoing unilateral vocal cord paralysis at the one-year follow-up that was deemed permanent palsy, and all these patients had normal serum calcium at three months.

The demographic composition of our study sample corresponds to the regional epidemiology of thyroid disease, showing a female predominance and a mean age in the fourth decade of life. This suggests that our results are generalizable to the local patient population who typically undergo thyroid surgery. The comparable baseline characteristics between our two groups strengthen the internal validity of the observed outcome differences [12,13].

The safety concerns associated with MB pertain to high-dose intravenous administration (3-7.5 mg/kg), which has been linked to serotonin syndrome and coronary vasoconstriction. These complications are not relevant to our low-dose topical technique [14,15]. However, surgeons should be aware that dye spillage may temporarily obscure the field, and the rapid parathyroid washout creates only a transient window for identification. These practical considerations notwithstanding, the safety profile of topical MB appears favorable, particularly when compared to conventional visual dissection alone, which can be inadequate in complex cases where RLN and parathyroid anatomy is variable [16].

The clinical implications of our study's findings are substantial. Injury to the RLN and hypoparathyroidism are two of the most significant complications in thyroid surgery that affect laryngeal function, airway function, and calcium homeostasis. A method that nearly eliminates the risk of RLN injury and preserves parathyroid function to a greater extent, as indicated by higher calcium levels, represents a substantial leap forward in protecting patient safety. This can be especially useful in training or in limited-resource environments without advanced neuromonitoring capabilities.

Nevertheless, the present study has some limitations. As a single-center study, our findings may not be fully generalizable to other surgical settings or patient populations. A major limitation was the lack of a formal record or objective assessment of potential local/systemic adverse effects associated with the use of MB spray in the surgical field. Further prospective clinical studies on these aspects would be desirable to establish a complete safety profile of this procedure.

## Conclusions

Intraoperative topical MB spray is a highly effective adjunct in thyroid surgery. It facilitates rapid and accurate identification of the RLN and PGs, with better preservation. This method has a lower risk of RLN injury and a higher postoperative serum calcium level. We recommend its use in daily practice for thyroid surgery to improve patient care.
